# Facile Stereoselective Reduction of Prochiral Ketones by using an F_420_‐dependent Alcohol Dehydrogenase

**DOI:** 10.1002/cbic.202000651

**Published:** 2020-10-23

**Authors:** Caterina Martin, Gwen Tjallinks, Milos Trajkovic, Marco W. Fraaije

**Affiliations:** ^1^ Molecular Enzymology Group University of Groningen Nijenborgh 4 Groningen The Netherlands

**Keywords:** Biocatalysis, deazaflavin, enantioselectivity, prochiral ketones, reduction

## Abstract

Effective procedures for the synthesis of optically pure alcohols are highly valuable. A commonly employed method involves the biocatalytic reduction of prochiral ketones. This is typically achieved by using nicotinamide cofactor‐dependent reductases. In this work, we demonstrate that a rather unexplored class of enzymes can also be used for this. We used an F_420_‐dependent alcohol dehydrogenase (ADF) from *Methanoculleus thermophilicus* that was found to reduce various ketones to enantiopure alcohols. The respective (*S*) alcohols were obtained in excellent enantiopurity (>99 % *ee*). Furthermore, we discovered that the deazaflavoenzyme can be used as a self‐sufficient system by merely using a sacrificial cosubstrate (isopropanol) and a catalytic amount of cofactor F_420_ or the unnatural cofactor FOP to achieve full conversion. This study reveals that deazaflavoenzymes complement the biocatalytic toolbox for enantioselective ketone reductions.

## Graphical abstract

**An alternative to NADH dependence**: An F_420_‐dependent alcohol dehydrogenase can be used to asymmetrically reduce prochiral ketones. Conveniently, using a cheap sacrificial cosubstrate (isopropanol), the same enzyme can regenerate the deazaflavin cofactor.

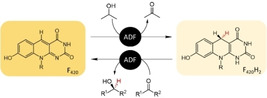

The biocatalytic production of chiral alcohols is feasible by employing different classes of enzymes: oxidoreductases (EC 1), hydrolases (EC 3), and lyases (EC 4).^[1]^ Several methods allow for the production of chiral alcohols and an effective method involves the enantioselective reduction of prochiral ketones by alcohol dehydrogenases (ADHs). ADHs have gained great interest due to their enantioselectivity, allowing for the development of many biotechnological applications.^[2]^ The applicability of ADHs is somewhat limited due to their cofactor dependence: most ADHs rely on the nicotinamide cofactors NADH or NADPH for performing reductions.^[3]^ Often, cofactor regeneration is achieved by using an enzyme‐coupled process.^[4]^ We explored another type of ADH: an F_420_‐dependent ADH. Such type of ADH had never been explored for biocatalytic reductions of ketones. The deazaflavin cofactor F_420_ was discovered in 1972, first in methanogens and later also in actinobacteria and other bacteria.[^5^, ^6^, ^7^] The redox active part of the cofactor resembles the isoalloxazine ring of the canonical flavin cofactors (Figure 1). The differences are the lack of the N5, different groups on the benzylic moiety, and the poly‐γ‐glutamate lactyl tail instead of the adenine part of FAD. The absence of N5 (a carbon instead) dictates that the cofactor is an obligatory hydride transfer agent, similar to the nicotinamide cofactors. The F_420_ cofactor displays an absorption maximum at 420 nm, hence its name. The redox potential of this redox cofactor is exceptionally low: −380 mV [(220 mV for FAD and −320 mV NAD(P)].[^5^, ^8^]

**Figure 1:**
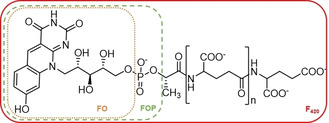
Structural formula of the F_420_ deazaflavin cofactor. The deazaflavins FO and FOP are also indicated.

While flavin and nicotinamide cofactors are readily available together with a huge number of enzymes utilizing these cofactors, the biocatalytic exploration of F_420_‐dependent enzymes is lagging behind. This is partly due to the fact that the deazaflavin cofactor was difficult to obtain. The deazaflavin cofactor can be isolated from F_420_‐producing microbes, such as *Mycobacterium smegmatis* or a F_420_‐producing recombinant microorganism.[^9^, ^10^] Although it involves a laborious process and yields small amounts, it is nowadays available for biocatalytic studies. Furthermore, we have recently demonstrated that a truncated version of the cofactor, lacking the poly‐γ‐glutamate lactyl tail (FOP, see Figure 1), is also accepted as cofactor by F_420_‐dependent enzymes.^[11]^ FOP represents an unnatural deazaflavin cofactor for which a relatively easy synthetic procedure has been developed. Another bottleneck for using F_420_‐dependent enzymes has been the lack of knowledge of and access to such biocatalysts. Particularly, these redox enzymes seem promising for performing selective reductions by virtue of the unique low redox potential deazaflavin cofactor. This would require regeneration of the reduced deazaflavin cofactor. This demand for F_420_H_2_‐regenerating enzymes has been satisfied by the recent discovery of several deazaflavoenzymes. The availability of several F_420_‐dependent glucose‐6‐phosphate dehydrogenases is especially attractive for this.^[12]^

In the last decade the interest in exploring deazaflavoenzymes for biocatalysis has increased.[^8^, ^13^, ^14^, ^15^] F_420_‐dependent enzymes were shown to be involved in the production of antibiotics, and could be used for the asymmetric reduction of prochiral imines and enones.[^16^, ^17^, ^18^, ^19^, ^20^] In this work we explored an F_420_‐dependent ADH (ADF) for its ability to perform enantioselective reductions of prochiral ketones. The F_420_‐dependent alcohol dehydrogenase from *Methanoculleus thermophilicus* was chosen for this. ADF is a relatively small enzyme (37 kDa) that was first described in 1989. It can be recombinantly produced in *Escherichia coli*, and its crystal structure has been elucidated.[^17^, ^21^] Previously it was shown that ADF converts small aliphatic secondary alcohols into ketones. It was shown that during this oxidation step a hydride is transferred in a stereoselective manner from the alcohol to the *si* face of the F_420_ cofactor (to the C5 atom).^[22]^ It was demonstrated that the enzyme is able to oxidize isopropanol to acetone and the reverse reaction.^[17]^ This was a clear indication that the enzyme may act as an enantioselective ketone reductase through an enantioselective hydride transfer.^[21]^

First, we explored the substrate scope of ADF to find out whether it is able to accept alcohols different from isopropanol. Alcohol oxidation activity could be easily monitored by measuring the decrease of absorbance of F_420_ (the reduced form does not absorb at 420 nm). ADF did not convert the following alcohols: heptan‐2‐ol, 5‐aminopentan‐1‐ol, 1‐aminopropan‐2‐ol, 3‐chloropropane‐1,2‐diol, 1‐phenylethane‐1,2‐diol, indan‐ol, acetoin, carveol, cyclohexylmethanol, tetrahydropyran‐2‐methanol, 3‐butyn‐2‐ol. Nevertheless, we could identify a set of aromatic and aliphatic alcohols that were not reported before as substrate of ADF. The corresponding ketones were investigated as prochiral substrates for ADF (Figure 2).

**Figure 2:**
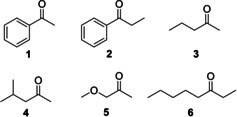
Ketones used for the biocatalytic exploration of ADF.

For the first test conversions, we used the F_420_‐dependent glucose‐6‐phosphate dehydrogenase (FGD) from *Rhodoccocus jostii* RHA1 (FGD) together with ADF (Figure 3a).^[12]^ Gratifyingly, it was found that the enzyme was able to fully and stereoselectively reduce methyl ketones to the corresponding (*S*) alcohols. For most ketones, excellent enantioselectivity was observed (>99 % *ee*), except for substrate 3 (92 % *ee*) and substrate 6 (racemic product; Table 1).

**Figure 3:**
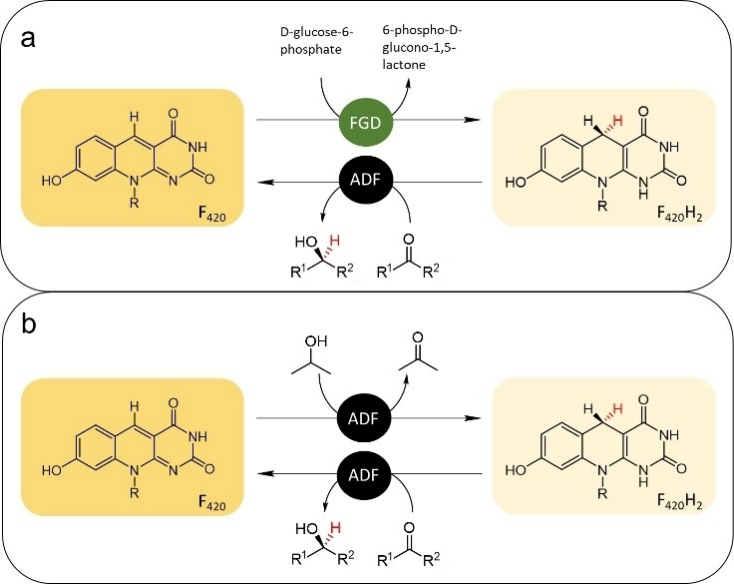
Coupled reactions for the reduction of prochiral ketones by ADF. ADF: F_420_‐dependent alcohol dehydrogenase, FGD: F_420_‐dependent glucose‐6‐phosphate dehydrogenase. a) d‐glucose‐6‐phosphate is used by FGD to regenerate F_420_H_2_. b) Self‐sufficient use of ADF for ketone reductions with isopropanol as cosubstrate to regenerate F_420_H_2_.

**Table 1:** Reduction of prochiral ketones by ADF.^[a]^

	Substrate	Cofactor	Cofactor regeneration^[b]]^	Conversion [%]	*ee* [%]
1EF_420_	**1**	F_420_	FGD	96	>99 (*S*)
1IF_420_	**1**	F_420_	–	95	>99 (*S*)
1IFop	**1**	FOP	–	5	n.d.
2EF_420_	**2**	F_420_	FGD	77	>99 (*S*)
2IF_420_	**2**	F_420_	–	80	>99 (*S*)
2IFop	**2**	FOP	–	3	n.d.
3EF_420_	**3**	F_420_	FGD	>99	92 (*S*)
3IF_420_	**3**	F_420_	–	>99	72 (*S*)
3IFop	**3**	FOP	–	94	>99 (*S*)
4EF_420_	**4**	F_420_	FGD	87	>99 (*S*)
4IF_420_	**4**	F_420_	–	93	>99 (*S*)
4IFop	**4**	FOP	–	2	n.d.
5EF_420_	**5**	F_420_	FGD	>99	>99 (*S*)
5IF_420_	**5**	F_420_	–	>99	>99 (*S*)
5IFop	**5**	FOP	–	>99	>99 (*S*)
6EF_420_	**6**	F_420_	FGD	>99	0
6IF_420_	**6**	F_420_	–	>99	0
6IFop	**6**	FOP	–	>99	0

[a] Values obtained using 250 mM sodium phosphate pH 7.0, 10 % glycerol, 1 % DMSO, 2.0 mM substrate, 2.0 mM ethyl benzene (internal standard), 20 μM ADF, 40 μM F420 or 40 μM FOP. [b] For cofactor regeneration, 20 mM glucose‐6‐phosphate with 10 μM FGD (“FGD”) or 200 mM isopropanol (“−”) was used. Details are given in the Supporting Information.n.d.: Conversions were too low for an accurate determination of *ee*.

In silico docking studies were performed to understand the molecular basis for the (*S*) stereoselectivity of ADF. For acetophenone (1) and methoxyacetone (5) docking suggested that the structurally favorable product is in both cases indeed the (*S*) enantiomer. This can be explained by examining the role and position of the active‐site residues. Previous studies postulated that His39, Trp43, Glu12 and Glu108 are crucial for substrate binding and catalysis.^[21]^ In the reduction reaction, a hydride transfer from the C5 atom of F_420_H_2_ to the ketone carbon with a simultaneous or stepwise proton addition from His39 to the ketone oxygen occurs. Hence, the specific orientation of substrate towards the F_420_ cofactor and His39 is decisive in the enantioselective outcome of the reaction. Docking of acetophenone revealed that Trp43 together with Val193, Trp229, Trp246, Cys249 and Phe255 form a hydrophobic pocket which snugly accomodates the aromatic ring of the substrate (Figure 4). As a result, acetophenone binds in the active site in such a way that only the (*S*) enantiomer can be formed: the hydride can only be transferred to the *re* face of the substrate. Positioning of acetophenone necessary to form the *R*‐enantiomeric alcohol is prevented because its aromatic ring cannot be accommodated in the active site in any other conformation. Docking of methoxyacetone resulted in an analogous optimal binding pose in which the apolar pocket next to the deazaflavin cofactor plays a crucial role in positioning the substrate such that hydride attack will occur on the *re* face of the substrate, assisted by proton transfer by His39. This will, again, only allow formation of (*S*)‐1‐methoxypropan‐2‐ol, as experimentally observed.

**Figure 4:**
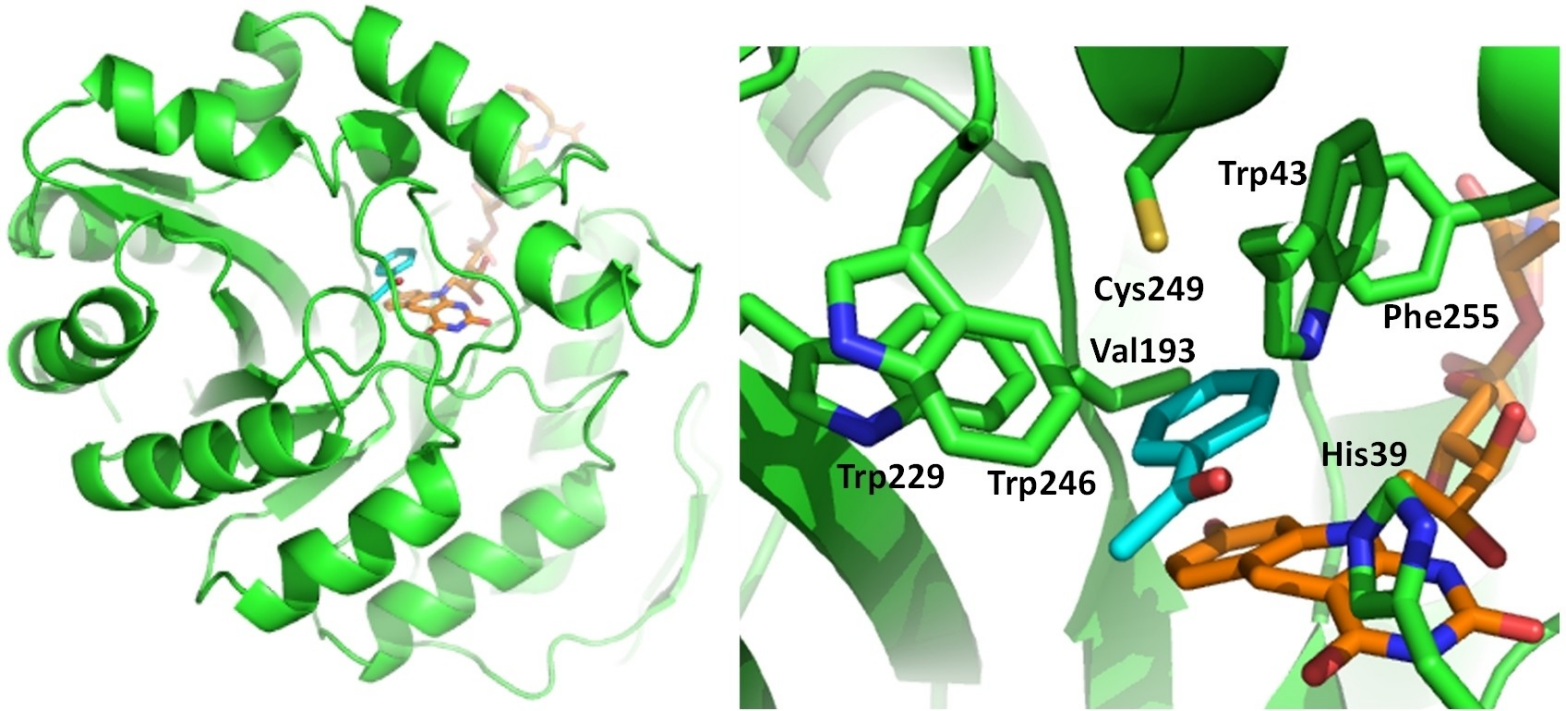
Binding pose of acetophenone (1) in ADF (PDB ID: 1RHC). Left: overall structure of ADF with docked substrate (cyan) and F_420_ (orange) highlighted in sticks. Right: close‐up of acetophenone binding.

The second aim of this work was to establish an efficient cofactor regeneration system. While the used glucose‐6‐phosphate dehydrogenase is effective in recycling F_420_H_2_, such cofactor regeneration system still requires an addition enzyme and a relatively expensive cosubstrate. Therefore, we explored whether ADF can be used as reductase and dehydrogenase in one pot, using a sacrificial and cheap alcohol as cosubstrate. This would eliminate the need for another enzyme for cofactor recycling. For this, we tested the use of isopropanol as it had been reported to be a good ADF substrate. In order to determine the highest concentration of isopropanol tolerated in conversions, we first assessed the thermostability of ADF in the presence of different amounts of isopropanol and its kinetic parameters with isopropanol. ADF was found to be relatively tolerant towards isopropanol with only a slight change in apparent melting temperature up to 200 mM isopropanol (*T*_m_ went from 57.5 to 56.0 °C, see Supporting Information). The steady‐state kinetic analysis confirmed that isopropanol is an effective substrate with a *K*_M_ value of 1.3 mM and a *k*_cat_ of 1.7 s^−1^ (see the Supporting Information). A concentration of 200 mM of isopropanol was selected to probe it as cosubstrate for the ADF‐catalyzed reduction of prochiral ketones. Remarkably, the use of isopropanol as cosubstrate worked extremely well. In fact, there was no difference between the use of glucose‐6‐phosphate dehydrogenase and glucose‐6‐phosphate or merely isopropanol for cofactor regeneration (Table 1). This shows that the use of isopropanol as coupled substrate is a valid and simple alternative to use ADF as enantioselective ketone reductase.

Finally, we also explored whether F_420_ can be replaced by an unnatural deazaflavin cofactor: FOP. We have recently shown that FOP can be prepared in a relatively easy manner and is often accepted by F_420_‐dependent enzymes.^[11]^ The results obtained using FOP as alternative cofactor with ADF and isopropanol as cosubstrate revealed that ADF can also operate with this alternative deazaflavin (Table 1). Yet, the use of FOP resulted in lower conversions for some of the ketones tested, while the enantioselectivity was largely retained. The inferior performance, when compared with F_420_, probably reflects a poor recognition of FOP by ADF.

In conclusion, the finding that ADF can reduce various prochiral ketones in a highly *S*‐stereoselective manner unveils a new biocatalytically relevant class of enzymes: F_420_‐dependent ketone reductases. They can be regarded as alternatives to nicotinamide cofactor‐dependent enzymes.^[23]^ Moreover, we demonstrate that isopropanol can be used as cheap cosubstrate for cofactor recycling, rendering ADF self‐sufficient. With the crystal structure of ADF available and many genes encoding for ADF homologues in the genome sequence database, it will be exciting to explore other variants for more demanding selective reductions.

## Experimental Section

Reagents and chemicals were purchased from Sigma‐Aldrich unless indicated otherwise. F_420_ was isolated from *Mycobacterium smegmatis* as described before.^[9]^ The production strain *M. smegmatis* mc^2^ 4517 was a kind gift from Dr. G. Bashiri (University of Auckland, New Zealand). The expression and purification of F_420_ dependent enzymes are described in the Supporting Information.

For ketone reductions, reaction mixtures contained 200 μL of 250 mM sodium phosphate pH 7.0, 10 % glycerol, 1 % DMSO, 2.0 mM substrate, 2.0 mM ethyl benzene (internal standard), 20 μM ADF, 40 μM F_420_ or 40 μM FOP, and 200 mM isopropanol or 20 mM glucose‐6‐phosphate with 10 μM FGD. The reaction was performed in a 1.5 mL Eppendorf tube in an Eppendorf Thermomixer at 25 °C and 500 rpm for 24 h. The reaction was extracted twice using 200 μL of ethyl acetate. The reactions passed over anhydrous magnesium sulfate and finally analyzed using GC (details in the Supporting Information).

## Conflict of interest

The authors declare no conflict of interest.

## Supporting information

As a service to our authors and readers, this journal provides supporting information supplied by the authors. Such materials are peer reviewed and may be re‐organized for online delivery, but are not copy‐edited or typeset. Technical support issues arising from supporting information (other than missing files) should be addressed to the authors.

Supplementary
