# Supplementary material for: Facile Stereoselective Reduction of Prochiral Ketones by using an F420‐dependent Alcohol Dehydrogenase
Source: Chembiochem. 2020 Oct 23;22(1):156–9. doi: 10.1002/cbic.202000651 (PMC7820951; doi:10.1002/cbic.202000651)
Supplement: Supplementary file 1 — Supplementary [file CBIC-22-156-s001.pdf]

ChemBioChem

Supporting Information

Facile Stereoselective Reduction of Prochiral Ketones by using an F_{420}-dependent Alcohol Dehydrogenase

Caterina Martin, Gwen Tjallinks, Milos Trajkovic, and Marco W. Fraaije*

Table of Contents

Expression and purification of F_{420}-dependent ADF and FGD 2
Steady state kinetics analyses 2
Isopropanol tolerance 3
Conversions 4
Substrate 1 4
Substrate 2 5
Substrate 3 6
Substrate 4 7
Substrate 5 8
Substrate 6 9
Substrate docking 10
References 10

Expression and purification of F_{420}-dependent ADF and FGD

An Adf-encoding gene fragment was ordered codon optimized for *Escherichia coli* and cloned into a pBAD to have the construct expressed with a C-terminal His-tag. Expression was performed using *Escherichia coli* NEB 10β. Cells carrying the plasmid were grown in Terrific broth (TB) supplemented with 50 µg mL^{-1} ampicillin until OD_{600} was 0.8 and expression was induced with L-arabinose 0.02% at 24 °C overnight. Cells were harvested by centrifugation at 5000 × g for 20 min at 4 °C. Cell pellets were resuspended in 250 mM sodium phosphate buffer pH 7.0 containing 1 mM phenylmethylsulfonyl fluoride and 10% v/w glycerol. The cells were lysed by sonication, using a Sonics Vibra-Cell VCX 130 sonicator with a 3 mm stepped microtip (5s on, 5s off, 70 % amplitude, 7 min). Cell debris were pelleted by centrifugation at 12000 × g for 20 min at 4 °C. The supernatant was applied to Ni-Sepharose High Performance (GE Healthcare) pre-equilibrated with 250 mM sodium phosphate pH 7.0, 10% v/w glycerol. The washing buffer was 250 mM sodium phosphate pH 7.0, 10% v/w glycerol, 10 mM imidazole and the elution buffer was 250 mM sodium phosphate pH 7.0, 10% v/w glycerol, 500 mM imidazole. The eluted protein was desalted using a desalting column pre-equilibrated with 250 mM sodium phosphate pH 7.0, 10% v/w glycerol. FGD was purified as previously described. [1] Purity of ADF and FGD was assessed with SDS-PAGE analysis and protein concentrations were measured by using the Bradford assay.

Steady state kinetics analyses

The employed assay measures the rate at which F_{420} is reduced (ε_{400} =25.7 $mM^{-1}cm^{-1}$). The buffer used was 250 mM sodium phosphate (pH 7.0), 10% v/w glycerol. For obtaining K_M and k_{cat} values, the data were fit using a regular Michaelis-Menten equation: $k_{obs}=k_{cat}*[S]/K_M + [S]$.

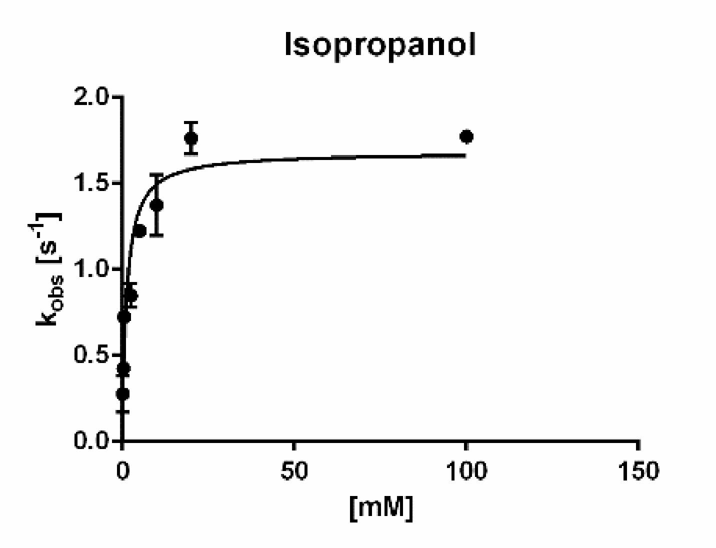

**K_M**
1.3±0.5 mM
k_{cat}
1.7±0.1 s^{-1}
***k_{cat}*/ K_M**
1.3 $s^{-1}mM^{-1}$

Isopropanol tolerance

The tolerance of ADF towards isopropanol was probed by measuring its apparent melting temperature by ThermoFluor. [2] Using a real-time PCR the temperature at which ADF unfolds in the presence of different concentrations of isopropanol was measured. For the measurements, 250 mM sodium phosphate pH 7.0, 10% glycerol, 1x Sypro Orange, and 10 µM ADF was used.

Table 1. Thermostability of ADF in the presence of isopropanol.

Isopropanol Concentration (mM)	T_m^{app} (°C)
50	57.5±0
100	57.5±0
200	56.0±0
500	53.5±0
1000	49.5±0

Conversions

Substrate 1

Analyzed using: Agilent Technologies 7890A GC system with column CP Chiralsil Dex CB (Agilent). Program: 40 °C to 130 °C in 5 min, hold 130 °C 10 min, 130 °C to 180 °C in 10 min, hold 180 °C in 5 min.

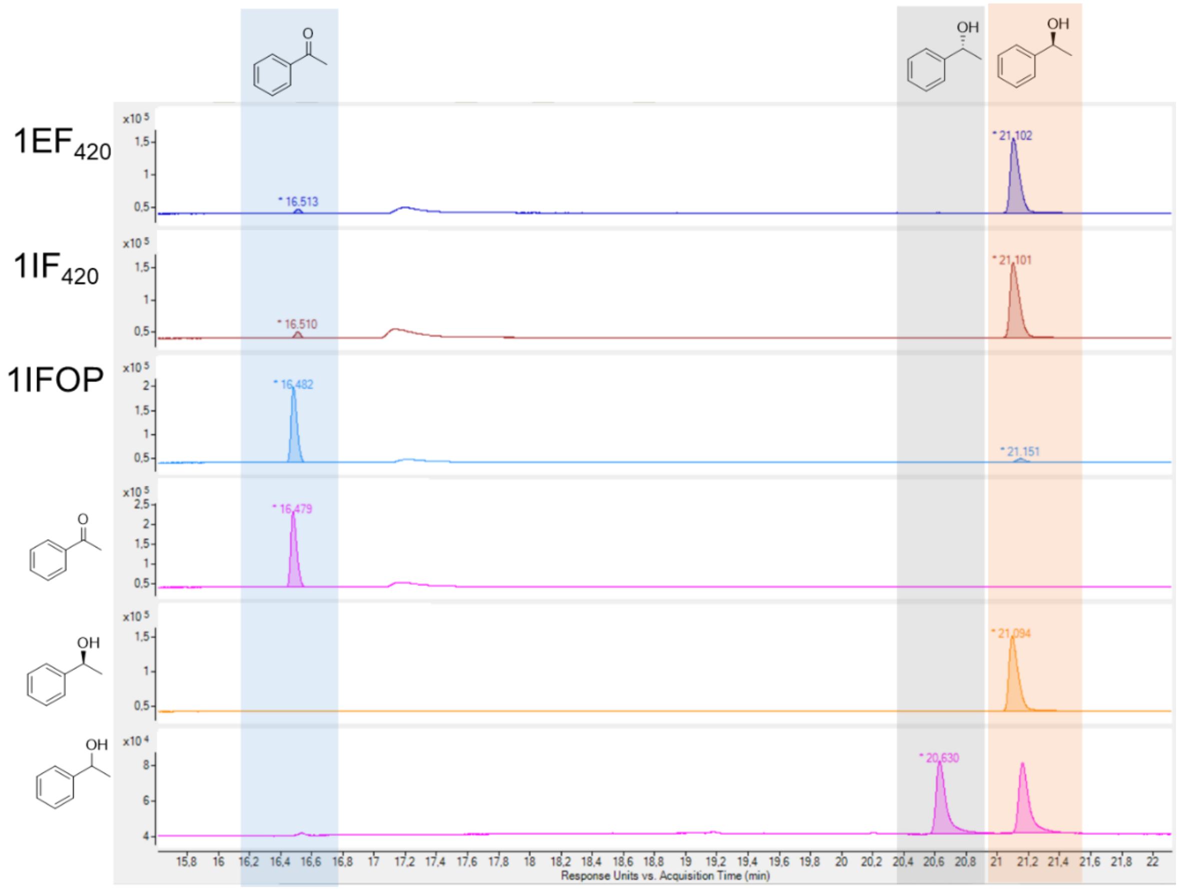

Substrate 2

Analyzed using: Agilent Technologies 7890A GC system with column CP Chiralsil Dex CB (Agilent). Program: 40 °C to 130 °C in 5 min, hold 130 °C 10 min, 130 °C to 180 °C in 10 min, hold 180 °C in 5 min.

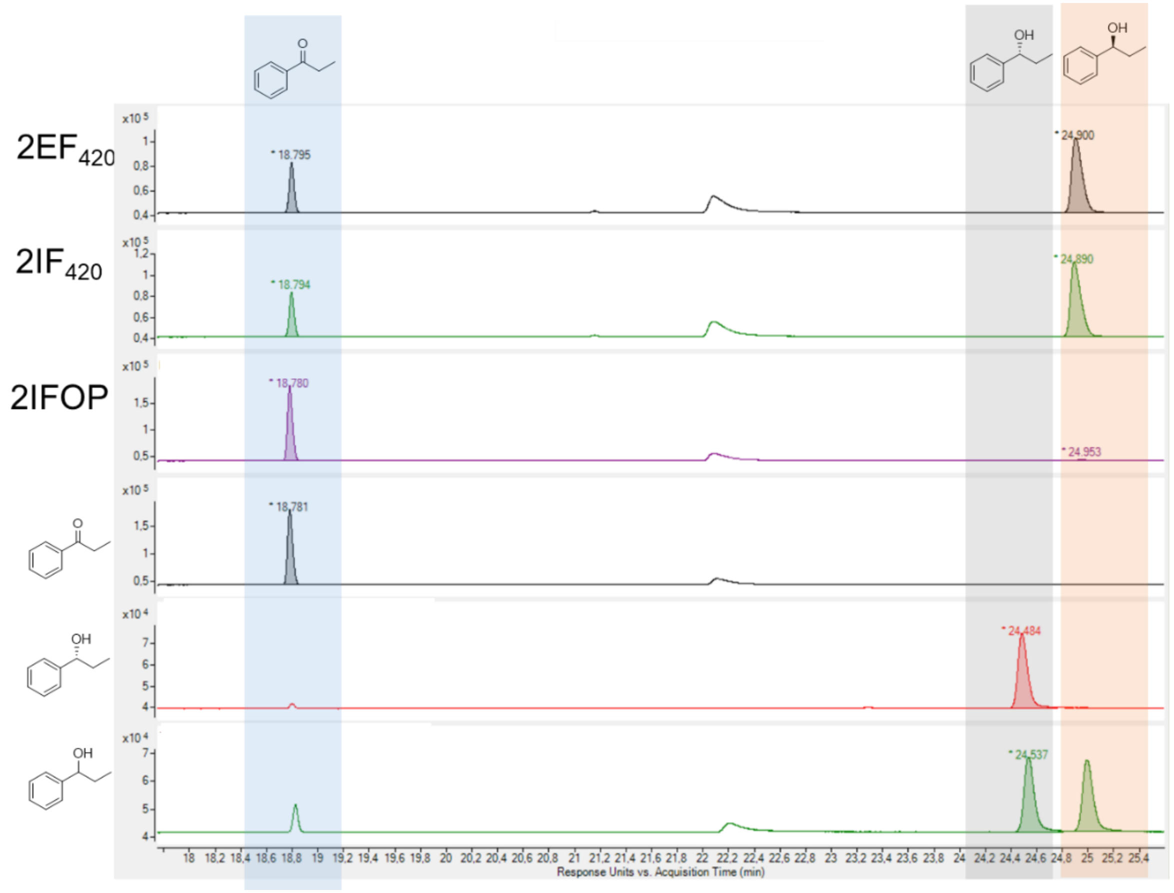

Substrate 3

Analyzed using: Agilent Technologies 7890A GC system with column CP Chiralsil Dex CB (Agilent). Program: 40 °C to 120 °C in 3 min, 120 °C to 40 °C in 10 min.

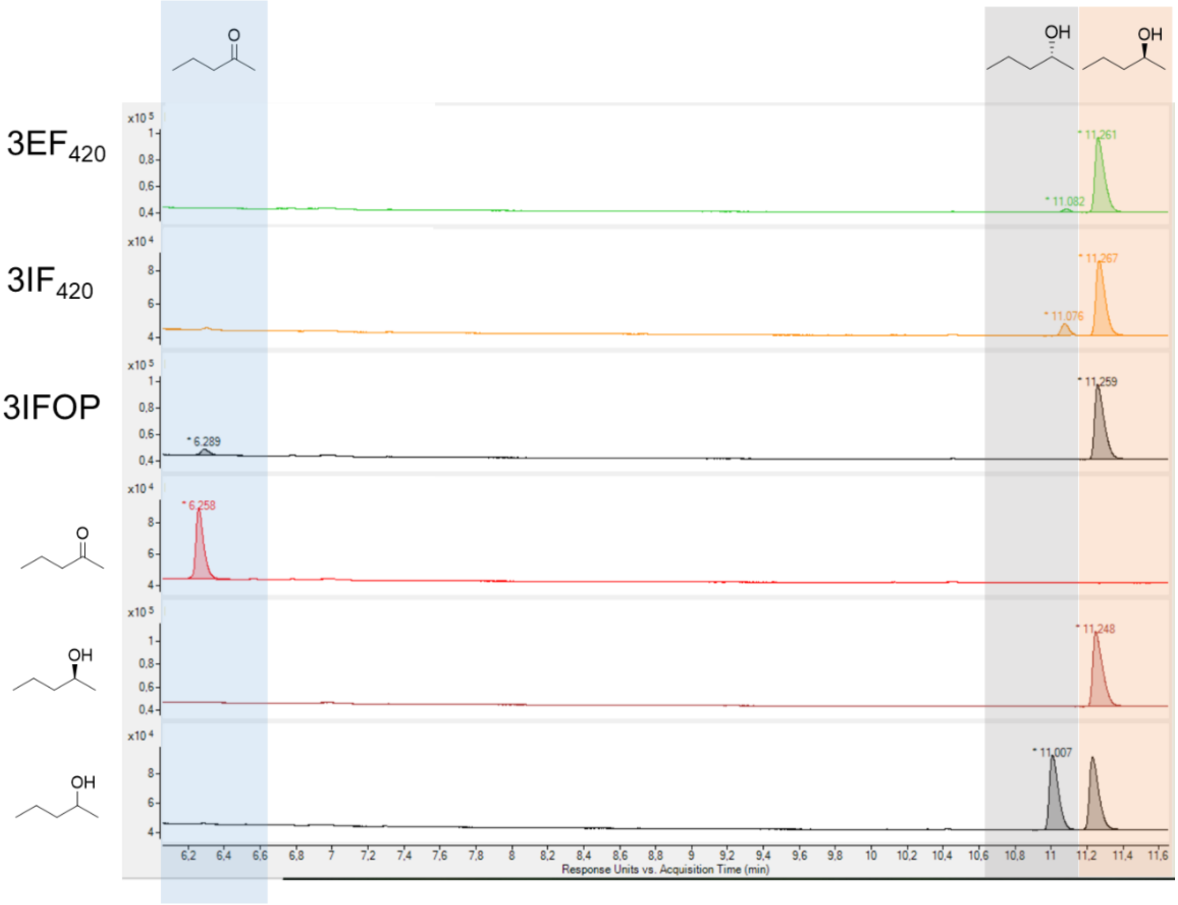

Substrate 4

Analyzed using: Agilent Technologies 7890A GC system with column CP Chiralsil Dex CB (Agilent).
Program: 40 °C to 140 °C in 5 min, 140 °C to 40 °C in 10 min.

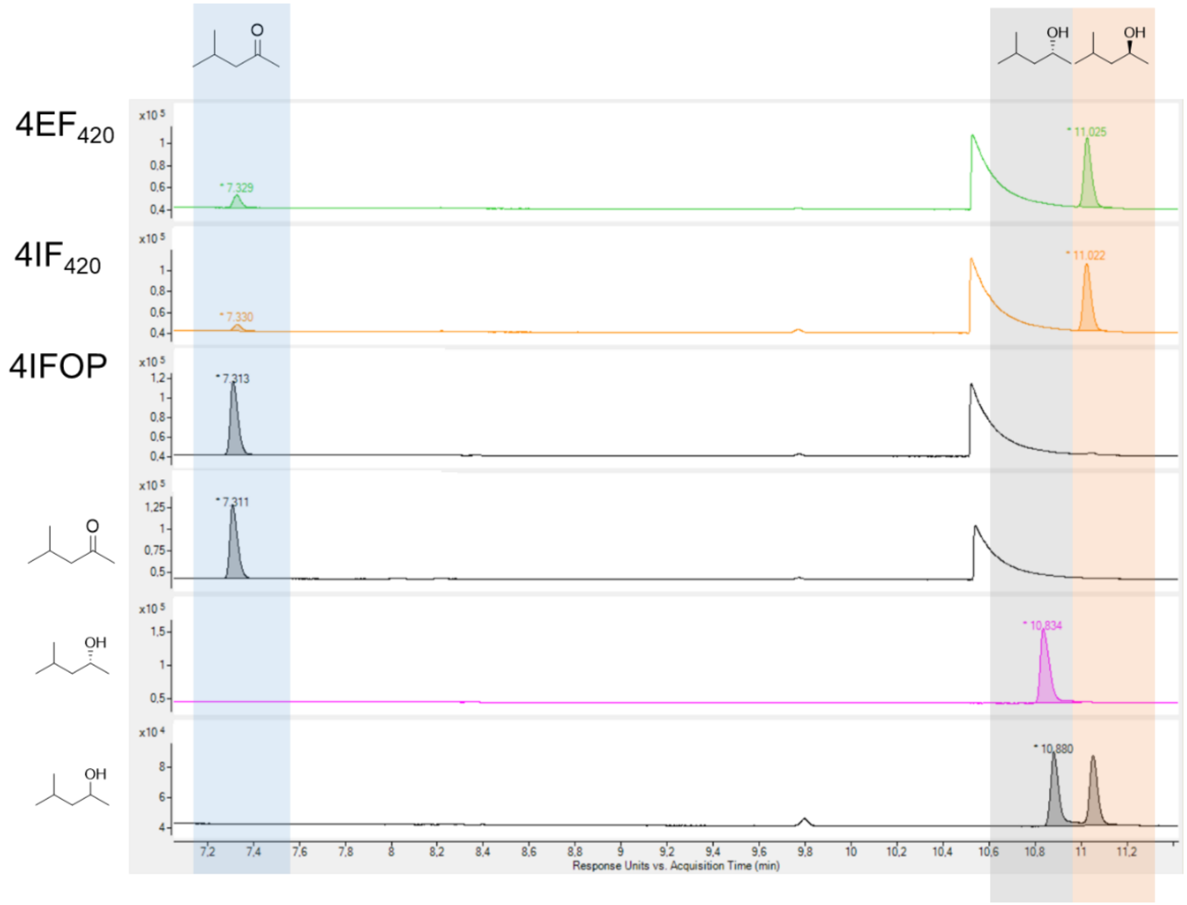

Substrate 5

Analyzed using: Agilent Technologies 7890A GC system with column FS-Hydrodex-B-TBDAc (Aurora Borealis) Program: 40 °C to 190 °C in 5 min, 190 °C to 40 °C in 10 min.

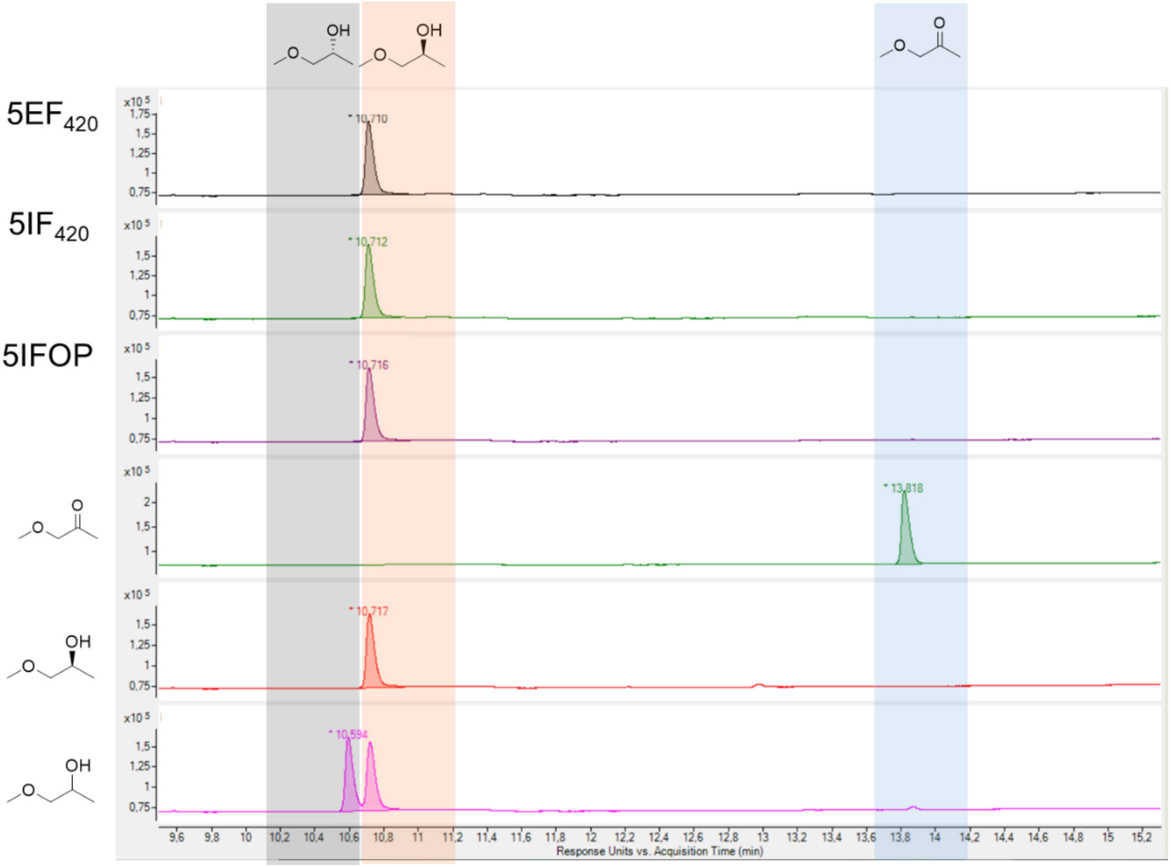

Substrate 6

Analyzed using: Agilent Technologies 7890A GC system with column FS-Hydrodex-B-TBDAc (Aurora Borealis) Program: 40 °C to 190 °C in 5 min, 190 °C to 40 °C in 10 min.

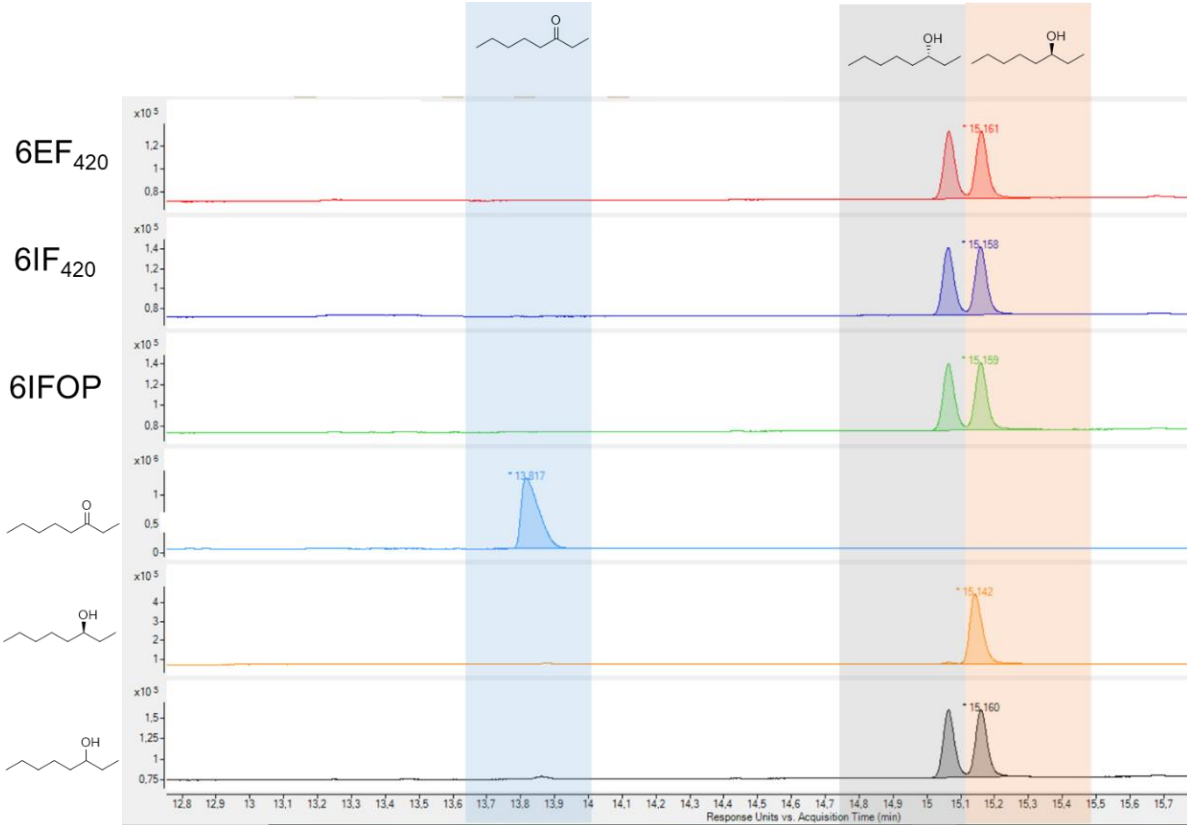

Substrate docking

Molecular docking was performed in YASARA Structure (version 19.12.14). [3] The crystal structure of ADF (1.8 Å resolution; PDB 1RHC [4]) was used. Substrates were built using YASARA, energy minimization was performed, and VINA was employed to perform the docking. [5] Docking was accomplished using the docking simulation macro 'dock_run.mrc' with a 5 Å cube cell size around the C5 atom of F_{420}, 100 runs, 2 Å cluster RMSD and using the YAMBER forcefield. [6] YASARA and UCSF Chimera were used to visualize the results. [7] Pymol was used for preparing the figures.

References

[1] Q. T. Nguyen, G. Trinco, C. Binda, A. Mattevi, M. W. Fraaije, *Appl. Microbiol. Biotechnol.* **2017**, *101*, 2831–2842.

[2] F. Forneris, R. Orru, D. Bonivento, L. R. Chiarelli, A. Mattevi, *FEBS J.* **2009**, *276*, 2833–2840.

[3] C. Guilbert, T. L. James, *J. Chem. Inf. Model.* **2008**, *48*, 1257–1268.

[4] S. W. Aufhammer, E. Warkentin, H. Berk, S. Shima, R. K. Thauer, U. Ermler, *Structure* **2004**, *12*, 361–370.

[5] O. Trott, A. J. Olson, *J. Comput. Chem.* **2012**, *32*, 174–182.

[6] E. Krieger, T. Darden, S. B. Nabuurs, A. Finkelstein, G. Vriend, *Proteins Struct. Funct. Genet.* **2004**, *57*, 678–683.

[7] E. F. Pettersen, T. D. Goddard, C. C. Huang, G. S. Couch, D. M. Greenblatt, E. C. Meng, T. E. Ferrin, *J. Comput. Chem.* **2004**, *25*, 1605–1612.
